# Vaginoscopic polypectomy for cervical polyps during pregnancy: an analysis of safety and influencing factors for pregnancy outcomes

**DOI:** 10.3389/fmed.2026.1780612

**Published:** 2026-03-31

**Authors:** Hui Zhao, Haixia Li, Zhikai Zhu, Baojun Yang, Wanli Gao, Limin Feng

**Affiliations:** 1 Department of Obstetrics and Gynecology, Beijing Tiantan Hospital, Capital Medical University, Beijing, China; 2 China National Clinical Research Center for Neurological Diseases, Beijing Tiantan Hospital, Capital Medical University, Beijing, China

**Keywords:** cervical polyp, miscarriage, polypectomy, preterm birth, vaginoscopy

## Abstract

**Objective:**

This study aimed to assess the safety of vaginoscopic polypectomy for managing cervical polyps during pregnancy and to identify factors influencing postoperative obstetric outcomes.

**Methods:**

We conducted a retrospective analysis of 99 pregnant patients who underwent vaginoscopic polypectomy for cervical polyps at Beijing Tiantan Hospital between January 2017 and February 2025. Based on pregnancy outcomes, patients were categorized into three groups: term delivery (*n* = 88), preterm delivery (*n* = 6), and spontaneous abortion (*n* = 5). Clinical characteristics were compared, and risk factors associated with pregnancy outcomes were analyzed. Patients were also classified into the cervical polyp group (*n* = 67) and the cervical decidual polyp group (*n* = 32) based on pathological diagnosis for further comparison of clinical features and prognosis.

**Results:**

All cervical polyps were resected successfully in a single procedure without any perioperative complications during pregnancy. The rates of term delivery, preterm delivery, and spontaneous abortion were 88.88% (88/99), 6.06% (6/99), and 5.05% (5/99), respectively. Preoperative vaginitis and postoperative recurrent bleeding differed significantly among the outcome groups (both *p* < 0.001) and were significantly associated with adverse pregnancy outcomes. Compared with the cervical polyp group, the decidual polyp group had a significantly greater polyp width, an earlier gestational age at diagnosis, and a higher rate of postoperative bleeding (*p* = 0.005, 0.013, and 0.036, respectively). However, the incidence of adverse pregnancy outcomes (preterm delivery and abortion) did not differ significantly between the two pathological groups (*p* > 0.05).

**Conclusion:**

In this observational clinical study, vaginoscopic polypectomy appeared to be a feasible and well-tolerated intervention for symptomatic cervical polyps during pregnancy. Preoperative vaginitis and postoperative rebleeding were identified as key factors associated with adverse obstetric outcomes. These findings highlight the importance of rigorous perioperative infection control and the prompt management of bleeding in this specific population. Further large-scale, prospective, controlled studies are warranted to confirm the procedure’s therapeutic effect and to validate the factors identified in this analysis.

## 1 Introduction

Cervical polyps, with a reported prevalence of 2%–5%, represent a common gynecological condition in reproductive-aged women (1). While clinical presentations range from vaginal bleeding and increased discharge to asymptomatic cases, their management during pregnancy presents a unique clinical challenge. The elevated estrogen and progesterone levels during gestation can stimulate rapid polyp growth, which may increase the risk of vaginal bleeding, secondary infection, and adverse pregnancy outcomes such as spontaneous abortion and preterm delivery (2).

The optimal management of cervical polyps during pregnancy remains controversial. Some studies suggest that polyps can induce recurrent bleeding and local inflammation, thereby altering the vaginal microenvironment and elevating the risk of chorioamnionitis and other adverse outcomes, and thus recommend early removal (3). Conversely, a conservative approach cautions against routine polypectomy, as the procedure itself may cause cervical irritation and potentially increase the risks of miscarriage and preterm delivery (4). This concern is particularly pertinent for the highly vascular and fragile decidual polyps commonly encountered during pregnancy (5, 6). Adding to this controversy, recent evidence suggests that antepartum polypectomy may not significantly increase the incidence of adverse pregnancy outcomes (7).

Traditional polypectomy techniques, including forceps grasping or loop electrosurgical excision, generally require speculum insertion and anesthesia and can present hemostatic challenges, often resulting in significant patient anxiety and discomfort. In contrast, vaginoscopy, as a “no-touch” technique, eliminates the need for a speculum and cervical clamp, avoids cervical dilation, and significantly reduces intraoperative pain without requiring anesthesia. These advantages contribute to superior patient tolerance and acceptance (8). Despite its potential benefits, systematic studies on the application of vaginoscopic polypectomy during pregnancy remain limited. Therefore, this study retrospectively analyzed the clinical data of 99 pregnant patients who underwent vaginoscopic polypectomy at our institution, with the aim of evaluating the safety of this technique and exploring the factors influencing postoperative pregnancy outcomes.

## 2 Materials and methods

### 2.1 Study population

A total of 99 patients who underwent vaginoscopic polypectomy for cervical polyps during pregnancy between January 2017 and February 2025 were enrolled from the Department of Gynecology at our institution.

Eligible patients had a singleton pregnancy with a confirmed cervical polyp and opted for surgical treatment due to recurrent vaginal bleeding. The exclusion criteria were as follows: (1) the presence of obstetric complications requiring pregnancy termination (e.g., severe preeclampsia, placental abruption, or placenta previa); (2) other serious medical or surgical conditions necessitating termination (e.g., congenital heart disease or appendicitis); and (3) existing cervical lesions or cervical insufficiency.

Participants were stratified postoperatively into three groups based on pregnancy outcome: spontaneous abortion (gestational age <28 weeks), preterm delivery (gestational age 28–36^+6^ weeks), and term delivery (gestational age ≥37 weeks). Furthermore, patients were categorized into two pathological subtypes—the cervical polyp group and the cervical decidual polyp group—according to the final histological diagnosis (9).

This study was approved by the Institutional Ethics Committee of our hospital (Approval No. KY2023-024-03).

### 2.2 Operative technique

In our clinical practice, polypectomy during pregnancy is recommended for polyps associated with recurrent bleeding, signs of infection, or features suspicious for malignancy. Following the completion of all necessary preoperative laboratory investigations to exclude surgical contraindications and after obtaining informed consent, patients diagnosed with vaginitis received standardized treatment based on cervical secretion analysis prior to the procedure. The procedure was performed only after infection resolution was confirmed through follow-up testing.

The procedure was performed using a 7.0-mm outer diameter bipolar resectoscope (Karl Storz, Tuttlingen, Germany). Continuous irrigation was maintained with 0.9% sodium chloride at a pressure of 80–100 mmHg and a flow rate of 300 mL/min. The power settings were 200 W for both cutting and coagulation.

Patients were positioned in the lithotomy position with a Trendelenburg tilt. The surgery was performed without anesthesia, and the patients remained conscious throughout the procedure. Without the use of a speculum or cervical tenaculum, the hysteroscope was inserted transvaginally under direct vision, advanced gently into the posterior fornix, and then rotated clockwise to systematically identify the external cervical os and the polyp stalk.

**Figure 1:**
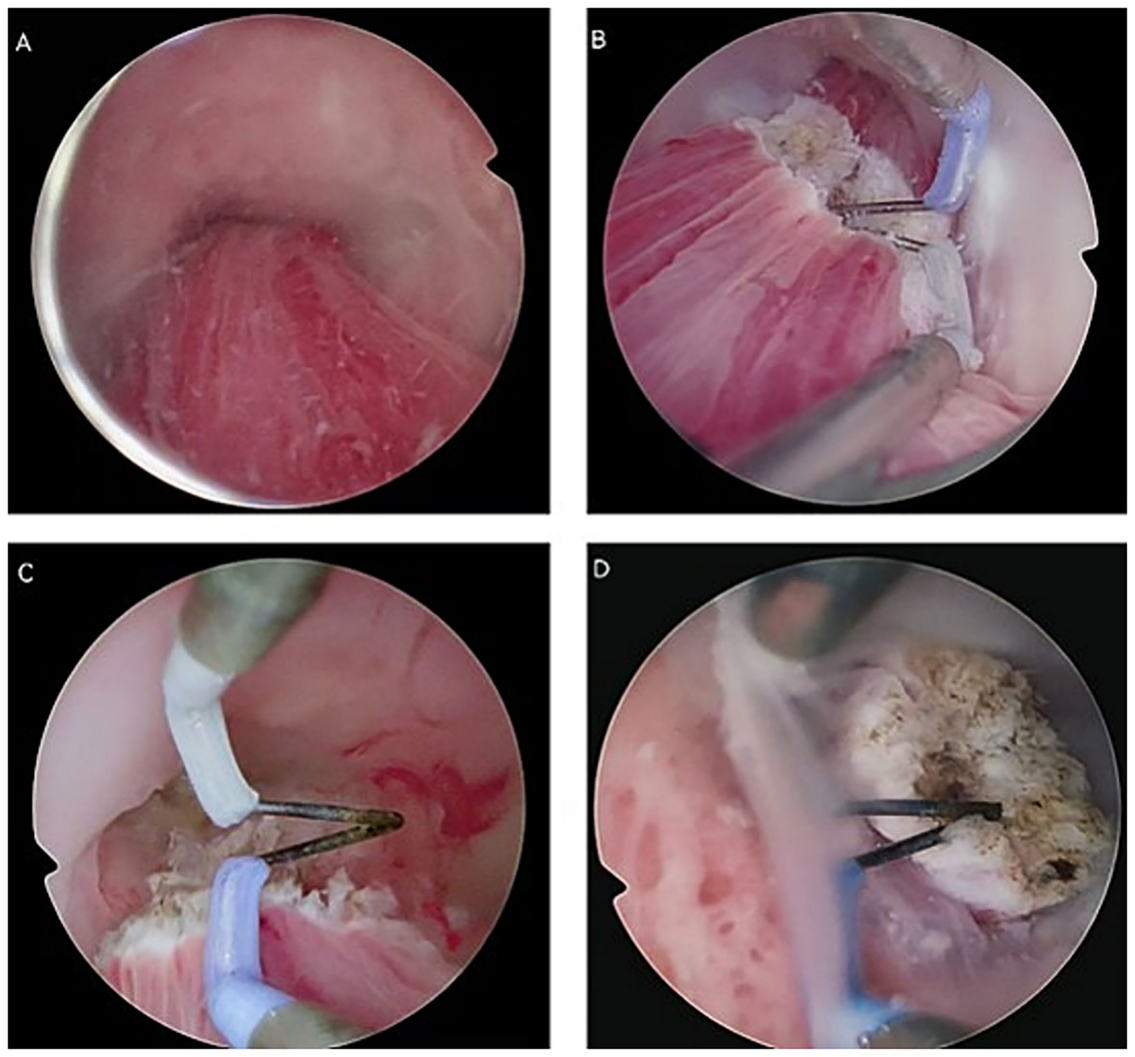
Basic operative procedures of vaginoscopic polypectomy in pregnant patients. **(A)** Identification of the external cervical os and the polyp. **(B)** Resection of the polyps at the external cervical os using a needle electrode. **(C)** Transection of the polyp using the “decapitation” technique. **(D)** Electrocoagulation of the residual polyp base to achieve hemostasis.

The polyp was resected at its base using the “decapitation” technique with either a needle or loop electrode. The resection site was then meticulously electrocoagulated to achieve hemostasis. In cases where the stalk was not fully accessible, the polyp was transected at the level of the external os, and the residual base was coagulated. To minimize intraoperative risks, the hysteroscope was not advanced deeply into the cervical canal, and a resection margin of at least 1 cm from the internal cervical os was maintained. Concurrent intraoperative ultrasound guidance was recommended during the procedure (Figure 1).

All resected specimens were further analyzed histopathologically. The procedures were exclusively performed by senior gynecological endoscopists. Postoperative pain was quantified using a visual analog scale (VAS). No prophylactic antibiotics or tocolytic agents were administered during the perioperative period.

### 2.3 Observation index

Data collected included (1) patient characteristics, including demographics, obstetric history, and mode of conception; (2) preoperative status, including bleeding patterns and results of vaginal discharge; (3) intraoperative measures, including blood loss, pain (VAS score), and complications; (4) surgical and pathological details, including gestational age at surgery, polyp dimensions (length and width), and definitive pathological diagnosis; and (5) postoperative outcomes, including rebleeding and comprehensive pregnancy outcomes, including miscarriage, preterm or term delivery, premature rupture of membranes (PROM), and neonatal data.

### 2.4 Statistical analysis

All statistical analyses were performed using R software (version 4.0.3). Continuous variables with a normal distribution were presented as the mean ± standard deviation and were compared using the *t*-test. Non-normally distributed data were summarized as median (Q1, Q3) and compared using the Wilcoxon rank-sum test. Categorical variables were summarized as frequency (percentage) and analyzed using the *χ*^2^ test or Fisher’s exact test, where applicable. A two-sided *p*-value of < 0.05 was considered statistically significant.

## 3 Results

### 3.1 Patient characteristics and pregnancy outcomes

A total of 99 patients were included in this study. The median age was 31 years (range, 29–35 years of age), and all patients presented with recurrent vaginal bleeding during pregnancy. Vaginoscopic polypectomy was successfully performed in a single session for all patients, with complete polyp resection achieved. The median gestational age at polyp detection was 12 weeks (range, 8–17 weeks). The median gestational age at surgery was 17 weeks (range, 15–21 weeks), and the median interval from bleeding onset to surgery was 5 weeks (range, 2–8 weeks). Gestational ages at diagnosis and intervention for patients in different outcome groups are shown in Table 1. The procedure was well-tolerated, with a median intraoperative visual analog scale (VAS) score of 1 (range, 0–1), indicating minimal to mild pain. The median estimated blood loss was 1 mL (range, 0.5–2 mL), and no intraoperative or postoperative complications occurred. Pathological examination identified 67 cases as cervical polyps and 32 cases as decidual polyps, with no malignant findings detected.

**Table 1:** Comparison of clinical features: Between miscarriage, preterm delivery and term delivery groups.

Clinical feature	Term delivery (*n* = 88)	Preterm delivery (*n* = 6)	Miscarriage (*n* = 5)	Sum (*N* = 99)	*p*-value
Age (years)^a^	31 (29, 35)	31 (29.5, 31.75)	32 (30, 32)	31 (29, 35)	0.944
Gravida (%)					0.219
1	61 (69.32%)	2 (33.33%)	3 (60%)	66 (66.67%)	
2	18 (20.45%)	4 (66.67%)	2 (40%)	24 (24.24%)	
3	8 (9.09%)	0 (0%)	0 (0%)	8 (8.08%)	
4	1 (1.14%)	0 (0%)	0 (0%)	1 (1.01%)	
Parity (%)					0.498
0	68 (77.27%)	4 (66.67%)	3 (60%)	75 (75.76%)	
1	18 (20.45%)	2 (33.33%)	2 (40%)	22 (22.22%)	
2	2 (2.27%)	0 (0%)	0 (0%)	2 (2.02%)	
IVF-ET (%)	1 (1.14%)	0 (0%)	0 (0%)	1 (1.01%)	1
Polyp length (cm)^a^	2 (2, 3)	2 (1.62, 2.75)	1.5 (1.5, 2)	2 (2, 3)	0.383
Polyp width (cm)^a^	1 (1, 2)	1 (1, 1)	1 (1, 1)	1 (1, 2)	0.506
Gestational weeks at polyp detection^a^	12 (8, 17)	14.5 (9, 17)	10 (8, 10)	12 (8, 17)	0.247
Gestational weeks at polypectomy^a^	18 (15, 21)	17 (15.5, 18.5)	16 (14, 17)	17 (15, 21)	0.437
Pathology (%)					0.159
Endocervical polyps	62 (70.45%)	2 (33.33%)	3 (60%)	67 (67.68%)	
Decidual polyps	26 (29.55%)	4 (66.67%)	2 (40%)	32 (32.32%)	
Vaginitis (%)	16 (18.18%)	5 (83.33%)	5 (100%)	26 (26.26%)	<0.001*
History of spontaneous abortion (%)	5 (5.68%)	1 (16.67%)	0 (0%)	6 (6.06%)	0.516
History of preterm birth (%)	0 (0%)	1 (16.67%)	0 (0%)	1 (1.01%)	0.111
History of cervical coning (%)	0	0	0	0	-
Intraoperative blood loss (ml)^a^	1 (1, 2)	0 (0, 3.75)	1 (1, 1)	1 (0.5, 2)	0.497
Bleeding after polypectomy (%)	2 (2.27%)	6 (100%)	5 (100%)	13 (13.13%)	<0.001*
Mode of delivery (%)					0.068
Vaginal delivery	58 (65.91%)	6 (100%)	5 (100%)	69 (69.7%)	
Cesarean section	30 (34.09%)	0 (0%)	0 (0%)	30 (30.3%)	
PROM (%)	13 (14.77%)	3 (50%)	0 (0%)	16 (16.16%)	0.08
Birth weight (kg)^a^	3290.74 ± 443.6	2,580 ± 533.7		3245.37 ± 479.49	<0.001*

a The data indicate the mean ± standard deviation or median (interquartile range);*p < 0.05; IVF-ET, in vitro fertilization and embryo transfer; PROM, preterm rupture of membranes.

Regarding pregnancy outcomes, the majority of patients (88/99, 88.88%) delivered at term. Spontaneous abortion and preterm delivery occurred in 5 (5.05%) and 6 (6.06%) patients, respectively. Premature rupture of membranes (PROM) occurred in 16 patients (16.16%).

### 3.2 Comparison of clinical characteristics among different pregnancy outcome groups

As shown in Table 1, significant differences among the three outcome groups (term delivery, preterm delivery, and spontaneous abortion) were observed in the prevalence of preoperative vaginitis, the incidence of postoperative rebleeding, and neonatal birth weight (all *p* < 0.001). Preoperative vaginitis was observed in 16 patients (18.18%) in the term delivery group, compared with five patients (83.33%) in the preterm delivery group and five patients (100%) in the spontaneous abortion group. Postoperative recurrent bleeding occurred in two patients (2.27%) in the term delivery group, whereas it occurred in all six patients (100%) in the preterm delivery group and all five patients (100%) in the spontaneous abortion group.

In contrast, maternal age, gravidity, parity, mode of conception, history of spontaneous abortion or preterm delivery, history of cervical coning, polyp dimensions (length and width), pathological type, gestational age at polyp detection or surgery, intraoperative blood loss, mode of delivery, and incidence of PROM did not differ significantly across the groups (all *p* > 0.05).

Further pairwise comparisons of the significant variables (Table 2) revealed that both preoperative vaginitis and postoperative rebleeding were significantly more frequent in the abortion and preterm delivery groups compared with the term delivery group (all *p* < 0.05).

**Table 2:** Comparison of clinical features: between miscarriage and term delivery groups, between preterm delivery and term delivery groups, between miscarriage and preterm delivery groups.

Clinical feature	Group	Group 1	Group 2	*p*-value
Vaginitis (%)	Term vs. preterm birth	16 (18.18%)	5 (83.33%)	0.001*
	Term vs. miscarriage	16 (18.18%)	5 (100%)	<0.001*
	Preterm birth vs. miscarriage	5 (83.33%)	5 (100%)	1
Bleeding after polypectomy (%)	Term vs. preterm birth	2 (2.27%)	6 (100%)	<0.001*
	Term vs. miscarriage	2 (2.27%)	5 (100%)	<0.001*
	Preterm birth vs. miscarriage	6 (100%)	5 (100%)	1

*p < 0.05.

### 3.3 Comparison between the cervical polyp and decidual polyp groups

Clinical characteristics were further compared based on pathological diagnosis (Table 3). The decidual polyp group exhibited significantly greater polyp width, an earlier gestational age at polyp detection, and a higher rate of postoperative bleeding compared with the cervical polyp group (all *p* < 0.05). However, no significant intergroup differences were found in maternal age, gravidity, parity, mode of conception, history of spontaneous abortion or preterm delivery, history of cervical coning, or polyp length. Although the decidual polyp group showed a tendency toward higher rates of spontaneous abortion, preterm delivery, and PROM, these differences were not statistically significant (all *p* > 0.05).

**Table 3:** Comparison of clinical features: between endocervical polyp and decidual polyp.

Clinical feature	Endocervical polyp (*n* = 67)	Decidual polyp (*n* = 32)	*p*-value
Age (years)^a^	31 (29, 34)	32.5 (30, 35.25)	0.170
Gravida (%)			0.107
1	49 (73.13%)	17 (53.12%)	
2	13 (19.4%)	11 (34.38%)	
3	5 (7.46%)	3 (9.38%)	
4	0 (0%)	1 (3.12%)	
Parity (%)			0.050
0	56 (83.58%)	20 (62.50%)	
1	10 (14.93%)	11 (34.37%)	
2	1 (1.49%)	1 (3.12%)	
IVF-ET (%)	1 (1.49%)	0 (0%)	1.000
Polyp length (cm)^a^	2 (1.5, 3)	2 (2, 3)	0.519
Polyp width (cm)^a^	1 (0.9, 1.5)	1.5 (1, 2)	0.013*
Gestational weeks at polyp detection^a^	13 (9.5, 18)	8.5 (8, 14)	0.005*
Gestational weeks at polypectomy^a^	19 (16, 21.5)	17 (15, 19)	0.110
Vaginitis (%)	17 (25.37%)	9 (28.12%)	0.771
History of spontaneous abortion (%)	5 (7.46%)	1 (3.12%)	0.692
History of preterm birth (%)	0 (0%)	1 (3.12%)	0.323
History of cervical coning (%)	0	0	—
Intraoperative blood loss (ml)^a^	1 (1, 2)	1 (0, 2)	0.565
Bleeding after polypectomy (%)	5 (7.46%)	8 (25%)	0.036*
Mode of delivery (%)			0.084
Vaginal delivery	43 (64.18%)	26 (81.25%)	
Cesarean section	24 (35.82%)	6 (18.75%)	
Gestational weeks at delivery	39 (38, 40)	39 (37, 39)	0.054
Preterm delivery (%)	2 (2.99%)	4 (12.5%)	0.160
Miscarriage (%)	3 (4.48%)	2 (6.25%)	1.000
PROM (%)	8 (11.94%)	8 (25%)	0.099
Birth weight (kg)^a^	3307.27 ± 463.25	3113.33 ± 494.6	0.067

a The data indicate the mean ± standard deviation or median (interquartile range); *p < 0.05; IVF-ET, *in vitro* fertilization and embryo transfer; PROM, preterm rupture of membranes.

## 4 Discussion

### 4.1 Clinical significance

Cervical polyps during pregnancy, particularly when manifesting as recurrent vaginal bleeding, often require differential diagnosis from threatened abortion. Misdiagnosis can lead to a substantial psychological burden for patients. Evidence indicates that pregnant individuals with cervical polyps face an approximately fourfold increased risk of intrauterine infection compared with those without polyps (2). Recurrent bleeding can disrupt the vaginal microecology, facilitating ascending infection and significantly increasing the risks of spontaneous abortion and preterm delivery (2). A large-scale study has further identified cervical polyps as an independent risk factor for late miscarriage and preterm birth (3). Histopathological analyses reveal significant infiltration of neutrophils and leukocytes within polyp tissues, which decreases significantly following polypectomy (10). Compared with conservative management, hysteroscopic polypectomy during pregnancy is associated with reduced rates of preterm birth (4.2% vs. 21.4%, *p* = 0.030), premature rupture of membranes (PROM) (18.8% vs. 45.2%, *p* = 0.025), and other pregnancy-related complications (7).

Nevertheless, the optimal management of cervical polyps in pregnancy remains a subject of clinical debate (9). Surgical intervention should be reserved for carefully selected cases, guided by a comprehensive assessment of the patient’s psychological state, duration of bleeding, infection risk, and potential impact on pregnancy outcomes. In our practice, resection is recommended during pregnancy for polyps associated with recurrent bleeding, signs of infection, or features suspicious for malignancy.

In the present study, all 99 enrolled patients presented with recurrent vaginal bleeding that persisted into the second trimester without spontaneous resolution and subsequently underwent vaginoscopic polypectomy. The procedure achieved a 100% success rate with no perioperative complications. The term delivery rate was 88.88%, with preterm delivery and spontaneous abortion rates of 6.06% and 5.05%, respectively. These outcomes favorably compare with those reported under conservative management (7), supporting the conclusion that vaginoscopic polypectomy is a safe, feasible, and minimally invasive option for managing symptomatic cervical polyps during pregnancy.

### 4.2 Advantages of vaginoscopy

Vaginoscopy is a “no-touch” technique that enables hysteroscopic procedures without a speculum or cervical tenaculum. By using a distension medium and the gentle dilating effect of the scope tip, the hysteroscope is introduced directly into the vaginal cavity for examination or intervention (8, 11). This approach significantly reduces patient anxiety and pain. In our study, the median visual analog scale (VAS) score was 1, demonstrating minimal procedural discomfort.

This technique obviates the need for anesthesia during pregnancy, thereby minimizing potential fetal exposure. Real-time ultrasound monitoring is routinely used, allowing conscious patients to visualize fetal cardiac activity, which may help alleviate psychological distress. The use of a bipolar electrosurgical system further enhances fetal safety, as the electrical current is confined to the loop and does not traverse the maternal body. Moreover, precise electrocoagulation under high-definition visualization ensures more effective hemostasis than traditional gauze compression, thereby significantly reducing the risk of postoperative rebleeding and infection.

### 4.3 Timing of intervention

Presently, there is no established consensus regarding the optimal gestational age for performing cervical polyp surgery during pregnancy. Due to studies suggesting an increased risk of adverse outcomes in the first trimester, it is generally recommended to schedule surgery after 12 weeks of gestation (5, 12). In our cases, the median operative gestational age was 17 weeks (range, 15–21 weeks). The median interval from the onset of bleeding to surgery was 5 weeks (range, 2–8 weeks), serving as a critical observation period to confirm the persistence of symptoms and establish definitive indications for the procedure.

### 4.4 The role of infection control

Genital tract infection is a well-established contributor to intra-amniotic inflammation and preterm birth (13, 14). In our study, the presence of preoperative vaginitis was significantly associated with adverse pregnancy outcomes following polypectomy (*p* < 0.001). Cervical polyps, as chronic inflammatory lesions, can cause ischemia, necrosis, and recurrent bleeding, which disrupt the vaginal microenvironment and promote bacterial proliferation. Conversely, persistent vaginitis may exacerbate local inflammation within the polyp, creating a potential “cervical polyp–vaginitis” cycle. Although identified infections were treated preoperatively, the high estrogen state during pregnancy promotes vaginal glycogen accumulation and alters local immunity, increasing susceptibility to recurrent vaginitis. Therefore, regular screening of vaginal secretions is recommended for pregnant patients with cervical polyps to enable the early detection and treatment of infections, which may help reduce the risk of adverse pregnancy outcomes and maintain a more stable local environment for prolonging gestation.

### 4.5 Management of postoperative bleeding

Persistent or recurrent bleeding during pregnancy elevates the risk of secondary infection, thereby increasing the likelihood of miscarriage and preterm birth. Our findings identified postoperative rebleeding as a factor significantly associated with adverse pregnancy outcomes (*p* < 0.001). Specifically, the incidence of postoperative bleeding was 2.27% in the term delivery group, compared with 100% in both the preterm delivery and spontaneous abortion groups. Persistent bleeding not only disrupts the local cervical environment and promotes ascending infection but may also act as a physicochemical stimulus for uterine contractions. Therefore, achieving effective hemostasis is paramount in the management of cervical polyps during pregnancy. Compared with traditional clamp-and-twist techniques, vaginoscopic polypectomy allows precise electrocoagulation under direct visualization, enabling simultaneous coagulation and cutting, which results in superior hemostasis. Nonetheless, heightened attention to, and systematic management of, postoperative bleeding remain crucial (15).

### 4.6 Considerations for decidual polyps

Decidual polyps, a histologically distinct subtype of cervical polyps that occur during pregnancy, can be differentiated from true cervical polyps (9). Whether they require surgical removal during pregnancy remains controversial. Some studies have suggested that the higher rate of adverse pregnancy outcomes following polypectomy may be associated with the pathological type, with decidual polyps posing a greater risk than true cervical polyps (16). Alternatively, other reports have attributed this risk to the inherent friability and hemorrhagic tendency of decidual tissue itself, which may predispose patients to adverse events (17).

In our study, compared with true cervical polyps, decidual polyps were detected at an earlier gestational age (median 8.5 weeks vs. 13 weeks, *p* = 0.005), exhibited a greater width (median 1.5 cm vs. 1 cm, *p* = 0.013), and were associated with a higher incidence of postoperative bleeding (25% vs. 7.46%, *p* = 0.036). These clinical characteristics are consistent with their underlying pathology: decidual polyps originate from decidualized tissue, often traverse the cervical canal, and disrupt the natural barrier function of cervical mucus, thereby increasing the risk of intrauterine infection. Their inherent friability and rich vascularity further contribute to their increased susceptibility to infection.

Nevertheless, no significant difference was observed in the incidence of adverse pregnancy outcomes between the two groups (*p* > 0.05). Consequently, clinical management should be guided primarily by the presence of symptoms—such as recurrent bleeding or signs of infection—rather than by pathological subtype. Since cervical polyps and decidual polyps cannot be distinguished based on appearance alone, care should be taken during the surgical procedure to avoid inserting the hysteroscope too deeply into the cervical canal. There is no requirement to completely remove the base of the polyp, as this helps prevent damage to the fetal membranes and placenta, thereby reducing the risk of adverse pregnancy outcomes. Therefore, active intervention for symptomatic decidual polyps is as safe and necessary as it is for true cervical polyps.

### 4.7 Limitations and future directions

This study has several inherent limitations. First, as a single-center retrospective analysis, its sample size is limited, particularly the number of adverse outcomes, which may introduce selection bias. Second, the analysis included only patients who underwent surgical intervention, without a conservative management control group or a direct comparison with traditional surgical techniques. In the future, large-scale, prospective, multivariable designs are warranted to further validate these findings and inform clinical practice.

## 5 Conclusion

In this observational clinical study, for pregnant patients with cervical polyps presenting with recurrent vaginal bleeding, anesthesia-free vaginoscopic polypectomy was found to be a feasible and well-tolerated intervention. It offers the advantages of minimal procedural pain, precise resection, and effective hemostasis. Optimizing pregnancy outcomes depends critically on a comprehensive perioperative strategy that includes vaginal infection control, meticulous intraoperative hemostasis, and prevention of postoperative bleeding.

## Data Availability

The original contributions presented in the study are included in the article/supplementary material; further inquiries can be directed to the corresponding author.

## References

[1] Tanos V Berry KE Seikkula J Abi Raad E Stavroulis A Sleiman Z . The management of polyps in female reproductive organs. Int J Surg. (2017) 43:7–16. doi: 10.1016/j.ijsu.2017.05.012, 28483662

[2] Wakimoto T Hayashi S Koh I Yamamoto R Ishii K. Relationship between unremoved cervical polyp in pregnancy and spontaneous preterm birth. Am J Obstet Gynecol. (2022) 227:899.e1–6. doi: 10.1016/j.ajog.2022.06.064, 35841937

[3] Hirayama E Ebina Y Kato K Akabane-Nakagawa K Okuyama K. Cervical polyps in early pregnancy are a risk factor for late abortion and spontaneous preterm birth: a retrospective cohort study. Int J Gynaecol Obstet. (2022) 156:64–70. doi: 10.1002/ijgo.13608, 33471369

[4] Tirlapur SA Adeyemo A O'Gorman N Selo-Ojeme D. Clinico-pathological study of cervical polyps. Arch Gynecol Obstet. (2010) 282:535–8. doi: 10.1007/s00404-010-1364-x, 20091045

[5] Fukuta K Yoneda S Yoneda N Shiozaki A Nakashima A Minamisaka T . Risk factors for spontaneous miscarriage above 12 weeks or premature delivery in patients undergoing cervical polypectomy during pregnancy. BMC Pregnancy Childbirth. (2020) 20:27. doi: 10.1186/s12884-019-2710-z, 31918700 PMC6953220

[6] Aoki S Hayashi M Seki K Hirahara F. Preterm premature rupture of membrane after polypectomy using an Endoloop polydioxanone suture II(™). Clin Case Reports. (2016) 4:331–2. doi: 10.1002/ccr3.503, 27099720 PMC4831376

[7] Zhang L Wang M Zhang S Han D Guo L Feng L. The efficacy and safety of cervical polypectomy with vaginoscopy in pregnant women. Arch Gynecol Obstet. (2024) 310:1945–50. doi: 10.1007/s00404-024-07583-2, 39103619

[8] Li H Yang B Gao W Huang C Li C Zhao H . Role of surgical vaginoscopy through no-touch hysteroscope in the treatment of female reproductive polyps. BMC Surg. (2024) 24:390. doi: 10.1186/s12893-024-02673-z, 39702228 PMC11657805

[9] Riemma G Della Corte L Vitale SG Cianci S La Verde M Giampaolino P . Surgical management of endocervical and decidual polyps during pregnancy: systematic review and meta-analysis. Arch Gynecol Obstet. (2023) 307:673–80. doi: 10.1007/s00404-022-06550-z, 35396972 PMC9984338

[10] Yoneda N Yoneda S Niimi H Ueno T Hayashi S Ito M . Polymicrobial amniotic fluid infection with mycoplasma/Ureaplasma and other bacteria induces severe intra-amniotic inflammation associated with poor perinatal prognosis in preterm labor. Am J Reprod Immunol. (2016) 75:112–25. doi: 10.1111/aji.12456, 26668114

[11] Smith PP Kolhe S O'Connor S Clark TJ. Vaginoscopy against standard treatment: a randomised controlled trial. BJOG. (2019) 126:891–9. doi: 10.1111/1471-0528.15665, 30801889

[12] Wang M Ye M Shen N Pan W Zhang H Wang X . Management of pregnant women with endocervical and decidual polyps: a systematic review and meta-analysis. Arch Gynecol Obstet. (2025) 312:375–84. doi: 10.1007/s00404-025-08056-w, 40407879 PMC12334433

[13] Kasprzykowska U Elias J Elias M Mączyńska B Sobieszczańska BM. Colonization of the lower urogenital tract with *Ureaplasma parvum* can cause asymptomatic infection of the upper reproductive system in women: a preliminary study. Arch Gynecol Obstet. (2014) 289:1129–34. doi: 10.1007/s00404-013-3102-7, 24318169 PMC3984420

[14] Tantengco OAG Menon R. Breaking down the barrier: the role of cervical infection and inflammation in preterm birth. Front Glob Womens Health. (2021) 2:777643. doi: 10.1093/infdis/171.6.1475, 35118439 PMC8803751

[15] Huang YL Chen RZ Le F. Analysis on pregnancy outcomes and risk factors of cervical polypectomy during the first and second trimester pregnancy. J Obstet Gynaecol Res. (2022) 48:2486–92. doi: 10.1111/jog.15352, 35801678 PMC13469927

[16] Tokunaka M Hasegawa J Oba T Nakamura M Matsuoka R Ichizuka K . Decidual polyps are associated with preterm delivery in cases of attempted uterine cervical polypectomy during the first and second trimester. J Matern Fetal Neonatal Med. (2015) 28:1061–3. doi: 10.3109/14767058.2014.942633, 25001427

[17] Zou J He Y Chen H Wang P Xiao X Liu S. A Clinicopathologic analysis of Decidual polyps: a potentially problematic diagnosis. Int J Clin Pract. (2022) 2022:2200790. doi: 10.1155/2022/2200790, 35685565 PMC9158793

